# CXCR4 Cardiac Specific Knockout Mice Develop a Progressive Cardiomyopathy

**DOI:** 10.3390/ijms20092267

**Published:** 2019-05-08

**Authors:** Thomas J. LaRocca, Perry Altman, Andrew A. Jarrah, Ron Gordon, Edward Wang, Lahouaria Hadri, Mark W. Burke, Georges E. Haddad, Roger J. Hajjar, Sima T. Tarzami

**Affiliations:** 1 Cardiovascular Research Center, Icahn School of Medicine at Mount Sinai, New York, NY 10128, USA; thomas.laRocca@mssm.edu (T.J.L.); perry.altman@mssm.edu (P.A.); rongordon@mssm.edu (R.G.); edward.wamg@mssm.edu (E.W.); Lahouaria.hadri@mssm.edu (L.H.); rogerhajjar@mssm.edu (R.J.H.); 2 Department of Medicine, Tufts University School of Medicine, Boston, MA 02111, USA; andrewjarrah@gmail.com; 3 Department of Physiology and Biophysics, College of Medicine, Howard University, Washington, DC 20060, USA; mark.burke@howard.edu (M.W.B.); ghaddad@howard.edu (G.E.H.)

**Keywords:** chemokine receptor-4 (CXCR4), CXCL12, cardiomyopathy, mitochondria

## Abstract

Activation of multiple pathways is associated with cardiac hypertrophy and heart failure. We previously published that CXCR4 negatively regulates β-adrenergic receptor (β-AR) signaling and ultimately limits β-adrenergic diastolic (Ca^2+^) accumulation in cardiac myocytes. In isolated adult rat cardiac myocytes; CXCL12 treatment prevented isoproterenol-induced hypertrophy and interrupted the calcineurin/NFAT pathway. Moreover; cardiac specific CXCR4 knockout mice show significant hypertrophy and develop cardiac dysfunction in response to chronic catecholamine exposure in an isoproterenol-induced (ISO) heart failure model. We set this study to determine the structural and functional consequences of CXCR4 myocardial knockout in the absence of exogenous stress. Cardiac phenotype and function were examined using (1) gated cardiac magnetic resonance imaging (MRI); (2) terminal cardiac catheterization with in vivo hemodynamics; (3) histological analysis of left ventricular (LV) cardiomyocyte dimension; fibrosis; and; (4) transition electron microscopy at 2-; 6- and 12-months of age to determine the regulatory role of CXCR4 in cardiomyopathy. Cardiomyocyte specific-CXCR4 knockout (CXCR4 cKO) mice demonstrate a progressive cardiac dysfunction leading to cardiac failure by 12-months of age. Histological assessments of CXCR4 cKO at 6-months of age revealed significant tissue fibrosis in knockout mice versus wild-type. The expression of atrial naturietic factor (ANF); a marker of cardiac hypertrophy; was also increased with a subsequent increase in gross heart weights. Furthermore, there were derangements in both the number and the size of the mitochondria within CXCR4 cKO hearts. Moreover, CXCR4 cKO mice were more sensitive to catocholamines, their response to β-AR agonist challenge via acute isoproterenol (ISO) infusion demonstrated a greater increase in ejection fraction, dp/dt_max_, and contractility index. Interestingly, prior to ISO infusion, there were significant differences in baseline hemodynamics between the CXCR4 cKO compared to littermate controls. However, upon administering ISO, the CXCR4 cKO responded in a robust manner overcoming the baseline hemodynamic deficits reaching WT values supporting our previous data that CXCR4 negatively regulates β-AR signaling. This further supports that, in the absence of the physiologic negative modulation, there is an overactivation of down-stream pathways, which contribute to the development and progression of contractile dysfunction. Our results demonstrated that CXCR4 plays a non-developmental role in regulating cardiac function and that CXCR4 cKO mice develop a progressive cardiomyopathy leading to clinical heart failure.

## 1. Introduction

Cardiovascular disease (CVD) continues to be the leading cause of death in the United States [1,2]. Hypertension, diabetes, hypercholesterolemia, and smoking are major risk factors for CVD promoting atherosclerosis, acute myocardial infarction, and congestive heart failure (CHF) [1,2,3]. CHF is a complex multifactorial syndrome leading to myocardial contractile dysfunction and reduced cardiac output [4,5]. While the development of cardiac dysfunction involves multiple distinct biochemical pathways, we are interested in small glycoproteins termed “chemokines”, one of the largest subclasses in the cytokine group. Chemokines signal through septahelical G-coupled receptors and elicit leukocyte migration to sites of an inflammatory or immune response and thus appear to play a pathogenic role in the development of CHF. Elevated circulating levels of these pro-inflammatory chemokines have been observed in patients with CHF for several decades [5]. Several therapeutic approaches have been designed to neutralize chemokines in hopes of reducing inflammatory cell migration and subsequent inflammation [6,7]. However these methods have not yet been commercially successful, due in part to the fact that the mechanisms underlying chemokine modulation of cardiac function are poorly understood [8].

Our group, alongside others, has shown that certain chemokines and their receptors are functional and constitutively expressed in cardiomyocytes [9,10,11]. The chemokine receptor, C-X-C Receptor 4 (CXCR4), and its ligand, C-X-C Ligand 12 (CXCL12), are necessary for the proper development of the embryonal heart. CXCR4 knockout, similar to the CXCL12 knockout, produces a lethal phenotype in mouse models around embryonic day 18.5 [12,13,14]. The CXCR4/CXCL12 axis has more recently been considered a possible therapeutic target for cardiovascular disease. In the adult heart, the CXCL12/CXCR4 chemokine axis plays a cardioprotective function by supporting progenitor cell chemotactic ingress into sites of myocardial ischemia [15]. CXCR4 mediated stem cell recruitment promotes cardiac myocyte survival and promotes a regenerative process in ischemic cardiomyopathies [15]. Interestingly, CXCL12 also has an alternative receptor, CXCR7 [16,17]; however, CXCR7 lacks a critical intracellular DRY domain in ICL-3 needed for G-protein activation, thus CXCR7 was originally thought to be either a scavenger for CXCL12 or a direct modulator of CXCR4 signaling [18]. It has now been determined in vitro using the HEK293 cell line that CXCR7 signals via β-arrestin 2 by activation of downstream Erk1/2 [19]. Recently we published distinct roles for CXCL12/CXCR4 and CXCL12/CXCR7 in the β-adrenergic response pathways in human cardiomyocytes [20]. Since β-ARs play a central role in heart failure pathogenesis via their modulation of calcium channel activity, a detailed analysis of the CXCL12/CXCR4 and/or CXCL12/CXCR7 relationship is warranted. 

We have previously demonstrated the physical and physiological association between β-AR and CXCR4 [9]. Hypertrophy and heart failure progression are associated with disturbances in the β-AR system and calcium cycling. Our present data indicate that CXCR4 cKO mice developed a progressive heart failure and die by 13 months of age. We postulated that the absence of CXCL12/CXCR4 led to an unchecked β-adrenergic response allowing for overactivation of adrenergic pathways, which ultimately contributed to the development and progression of contractile dysfunction. 

Catecholamine-induced cardiac hypertrophy and failure is associated with reduced contractile response to adrenergic agonists, an effect attributed to (1) the downregulation of myocardial β-ARs; (2) the uncoupling of β-ARs and adenylate cyclase and; and (3) the downstream modifications in cAMP-mediated signaling. We have recently demonstrated that CXCL12 treatment prevented isoproterenol-induced hypertrophy and interrupted the calcineurin/NFAT pathway in isolated adult rat cardiac myocytes [21]. Given these previous data, this study was designed to examine the significance of CXCR4 in cardiac function and assess the consequence of CXCR4 cKO on cardiac structure and function over the life of the animals. 

## 2. Results

### 2.1. CXCR4 Cardiac-Specific Knockout (CXCR4-cKO) Mice Develop a Progressive Cardiomyopathy Leading to Clinical Heart Failure

**Figure 1:**
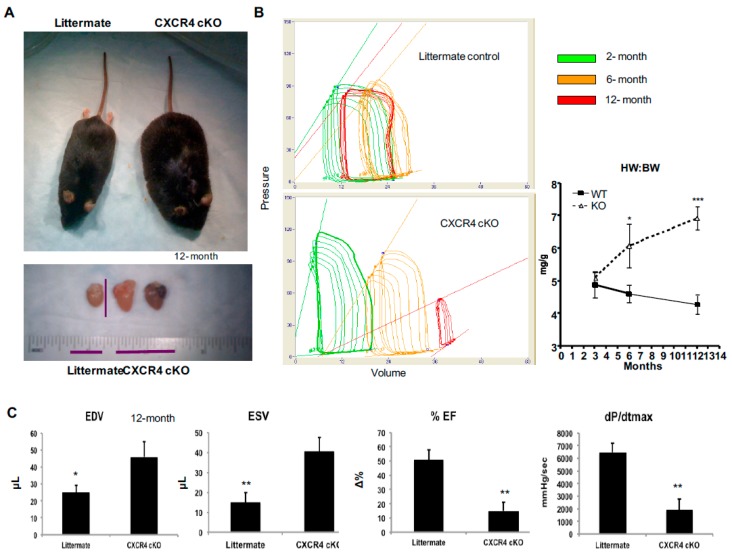
CXCR4 cardiac specific knockout (cKO) phenotype. We have successfully crossed the CXCR4^flox/flox^ (CXCR4^fl/fl4^) mice that possess both CXCR4 alleles with flox sites flanking CXCR4 exon 2 with the α-MHC Cre (Cre^+/0^) mice. (**A**) During our observational period, we noticed that study mice were very sick by the age of 12 months and that most died by 12–14 months. Phenotypic analyses of CXCR4 cKO at 12 months of age revealed significant peritoneal fluid collection, which correlated with an increase in the heart weight to body weight ratio. (**B**) In vivo hemodynamic data were acquired using a pressure-volume conductance catheter via an open-chest approach on mice aged 2- (green), 6- (orange), and 12-months (red) of ages are shown at baseline. Pre-load reduction studies were carried out by transiently occluding the inferior vena cava. Heart weight body weight ratio was assessed at 2-, 6-, and 12-months of age. (**C**) In vivo hemodynamic data were acquired and analyzed using 10×2 software, as demonstrated here. One-way ANOVA with Tukey’s post-hoc test was performed between all groups at each time point (n = 5–7 mice/group).

We developed this 12-month study to better understand the cardiac abnormalities, which underpin the development and progression of contractile dysfunction. Cardiac specific CXCR4 knockout mice were bred as has been previously published using a Cre-LoxP system (αMHC-CRE^+/0^;CXCR4^fl/fl^) [22]. Cardiac specific CXCR4 cKO was required as whole body deletion of CXCR4 results in a lethal mutation secondary to hematopoietic and cardiac defects [12]. We have previously shown that cardiomyocyte-specific deletion of CXCR4 produces viable mice [22,23], which we independently confirmed in this study. CXCR4 knockout was confirmed via RNA and protein studies as was demonstrated in our prior publication [22]. Following these animals over the 12-month study period has allowed us to demonstrate the development of a progressive cardiomyopathy as the animals aged; CXCR4 cKO hearts demonstrated abnormalities in both structure and contractile function (Figure 1). Reduced cardiac function in the experimental animals led to a significant peritoneal fluid which correlated well with an increase in the heart weight to body weight ratio (Figure 1A,B). Heart weight body weight ratio was assessed at 2-, 6-, and 12-months of age. CXCR4 cKO animals did not display structural or functional abnormalities at early time points between 2- and 3-months of age [22]. By 6-months; however, CXCR4 cKO animals had a 15% increase (* *p* < 0.05) in the heart weight:body weight (HW:BW ) ratio. By 12-months of age, CXCR4 cKO mice demonstrated a 50% increase (*** *p* < 0.001) in HW:BW which also correlated well with: (1) a 3-fold decline (* *p* < 0.05) in the maximum rate of change in left ventricular pressure (dp/dt_max_), an indicator of cardiac contractility (ionotrophy); and (2) a ≥2.5-fold decline (** *p* < 0.01) in ejection fraction (EF), and indicator of left ventricular pump function, as compared to littermate controls (Figure 1C). Additional data collection in mice aged greater than 12 months was not possible as most CXCR4 cKO mice died between the ages of 12–14 months. Hemodynamic evidence of increased left ventricular end-diastolic (EDV), increased end-systolic volume (ESV), and decreased ejection fraction (Figure 1C) further suggest global pump dysfunction secondary to myocardial hypertrophy (* *p* < 0.05, (** *p* < 0.01). These findings provide additional evidence that there is significant, ongoing cardiac remodeling in CXCR4 cKO mice hearts and that this remodeling likely drives the longitudinal effects of the CXCR4/CXCL12 axis on cardiac dysfunction and progression to clinical heart failure.

### 2.2. Abnormal Histopathological Features Were Evident in CXCR4 cKO Heart

**Figure 2:**
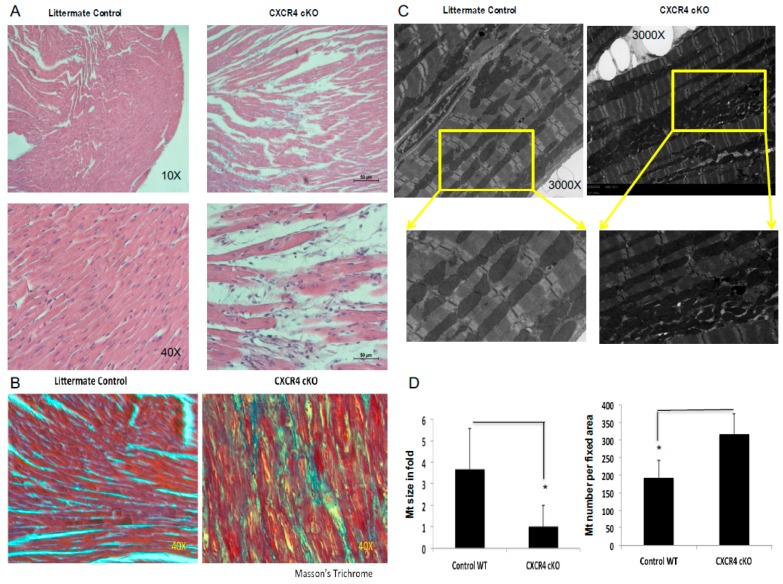
Histological assessment of CXCR4 cKO hearts. (**A**) We used conventional hematoxylin and eosin (H&E). Histological staining was done on left ventricular sections at the mid-papillary level and hearts were examined at low (10×) and high (40×) magnification. Images were captured via light microscopy at 40× magnification and a composite image of each sample was constructed. CXCR4 cKO hearts revealed significant structural abnormalities i.e., cardiac myocyte hypertrophy and the presence of cellular infiltrates at 6-months of age. (**B**) Masson’s Trichrome staining was used to assess for interstitial fibrosis. (**C**) Transition electron microscopy images were taken at 3000–5000× magnifications. Random pictures of each section were taken at 3000–5000× magnifications to count the number of mitochondria and at 1000× magnification to count the number of dark and light fibers. (**D**) After images were acquired, they were analyzed using ImageJ in order to count the number of mitochondria and to determine their mean size. Quantitative analyses of mitochondrial morphology revealed that CXCR4 cKOs have significantly higher counts of mitochondria. There was also significant difference in the size of mitochondria between the two genotypes as mitochondria were much smaller in the CXCR4 cKO group than in the wild-type population (* *p* < 0.05).

To better characterize cardiac structural abnormalities at the cellular and sub-cellular level in CXCR4 cKO animals, we used conventional hematoxylin and eosin (H&E) and Masson’s trichrome staining to assess for interstitial fibrosis. Histological staining was done on left ventricular (LV) sections at the mid-papillary level and hearts were examined at low (10×) and high (40×) magnification. Images were captured via light microscopy at 40× magnification and a composite image of each sample was constructed (Figure 2A,B). No abnormal histopathological features were evident at the 2-month time point [22]. However, by 6-months of age, CXCR4 cKO hearts revealed significant cardiac myocyte hypertrophy, the presence of cellular infiltrates, and progressive interstitial/peri-vascular fibrosis as compared with age-matched wild-type controls (Figure 2A,B and Figure S1). Transition electron microscopy was performed to further assess the subcellular abnormalities in the cardiomyocytes of CXCR4 cKO mice hearts at 6-months of age. Images were taken at 3000–5000× magnification (Figure 2C). Quantitative analyses of mitochondrial morphology revealed that CXCR4 cKOs have a significantly higher numbers of mitochondria. There was also a significant difference in the size of mitochondria between the two genotypes, as mitochondria were much smaller in the CXCR4 cKO group than in the wild-type population (Figure 2D).

Mitochondrial are the powerhouses of the cell and they have a high turnover rate. It has been well established that mitochondrial goes through the process of division (fission). Interference with such a division process could result in enlarged mitochondria, either because of the fact that these organelles continue to grow or the fact that mitochondrial organelles are also known to fuse (fusion), or some combination of the two. We have previously published that CXCR4 cKO heart shows dramatic decline in cytochrome c oxidase IV as compared to its littermate control [22]. Thus, a dramatic increase in mitochondria possibly could be the result and it acts as a compensatory mechanism for a reduction in cytochrome *c* oxidase and ATP synthase in these mice hearts. 

### 2.3. CXCR4 cKO Mice Have Significant Baseline Defects in Myocardial Function Beginning at 4-Months of Age as Assessed by MRI and In Vivo Hemodynamics

**Figure 3:**
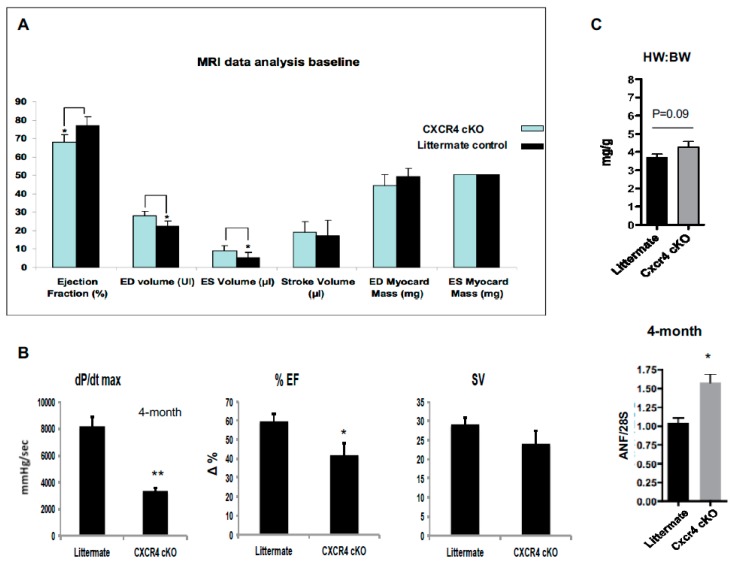
MRI data and hemodynamic assessment reveal a mild cardiomyopathy as early as 4 months of age. (**A**) We observed significance differences in hemodynamic parameters between CXCR4 cardiac deficient mice and wild-type controls. Over time, CXCR4 cKO mice exhibited a significant decline in systolic pressures, ejection fraction, and dp/dt_max_ as compared to controls (*n* = 5–7 mice/group). (**B**) In vivo hemodynamic data were acquired using a pressure-volume conductance catheter via an open-chest approach. Pre-load reduction studies were carried out by transiently occluding the inferior vena cava. We observed significance differences in baseline hemodynamics between CXCR4 cardiac deficient mice and wild-type controls as early as 4-months of age. In comparison of the two age groups, CXCR4 cKO mice exhibited a significant decline in contractility as indicated by the maximum rate of change in left ventricular pressure (dp/dt_max_) and reduced in ejection fraction (EF). as compared to littermate controls (*n* = 5–7 mice/group). Stroke volume did not change (* *p* < 0.05, ** *p* < 0.01). (**C**) At 4 months of age, HW:BW ratio (upper panel) shows a nonsignificant (*p* = 0.09) increase. We also assessed the mRNA levels of atrial naturietic factor (ANF), a well-known stress marker for hypertrophy (lower panel). There was significant ANF gene expression in the tissue generated from CXCR4 cKO heart. The levels of ANF mRNA were found to be 1.5× that of littermate controls (*n* = 5 mice/group) (* *p* < 0.05).

In order to better assess the onset of structural and functional changes CXCR4 cKO hearts, we used non-invasive murine gated cardiac magnetic resonance imaging (MRI) to better characterize the heart muscle itself at 4-months of age. This time point was chosen as mice were beginning to show significantly elevated levels of atrial natriuretic aactor (ANF), a well characterized marker of cardiac hypertrophy (Figure 3C), despite only a 10% increase (*p* = 0.09) in the HW:BW ratio (Figure 3C; upper panel). ANF levels were assessed via mRNA expression and it was found to be 1.5× that of littermate controls (Figure 3C; lower panel), supporting the notion that the molecular foundation for cardiac hypertrophy and subsequent heart failure are being laid prior to gross structural changes. MRI demonstrated that the CXCR4 cKO group had a 10% decrease in ejection fraction (CXCR4 68.04% vs littermate control 79.41%; * *p* < 0.05) as well as an increase in end-diastolic and end-systolic volumes suggesting mild functional defects (Figure 3A; Table 1). These changes did not translate into a reduction in stroke volume or cardiac output. Note that the heart rate was not significantly affected in the CXCR4 cKO mice (Table 1).

To further support our MRI data, invasive hemodynamic assessment was utilized. Hemodynamic analyses were performed on mice at 4-months of age to corroborate the imaging evidence (Figure 3B) (Table 2). In vivo hemodynamic data were acquired using a pressure-volume conductance catheter via an open-chest approach. Pre-load reduction studies were carried out by transiently occluding the inferior vena cava. We observed significance differences in baseline hemodynamics between CXCR4 cardiac deficient mice and wild-type controls (Figure 3B). CXCR4 cKO mice exhibited significant declines in both contractility and ejection fraction: (1) A 2.5× decrease (** *p* < 0.01) in contractility, as indicated by the maximum rate of change in left ventricular pressure (dp/dt_max_); and (2) a significant decrease (* *p* < 0.05) in ejection fraction as compared to littermate controls, indicating mild, but progressive heart failure (Figure 3B). Note that both MRI and hemodynamic data failed to show significant changes in stroke volume, suggesting that there was no significant remodeling in the CXCR4 cKO hearts at 4-months.

### 2.4. CXCR4 cKO Mice Are More Sensitive to an Acute Isoproterenol Challenge In Vivo

**Figure 4:**
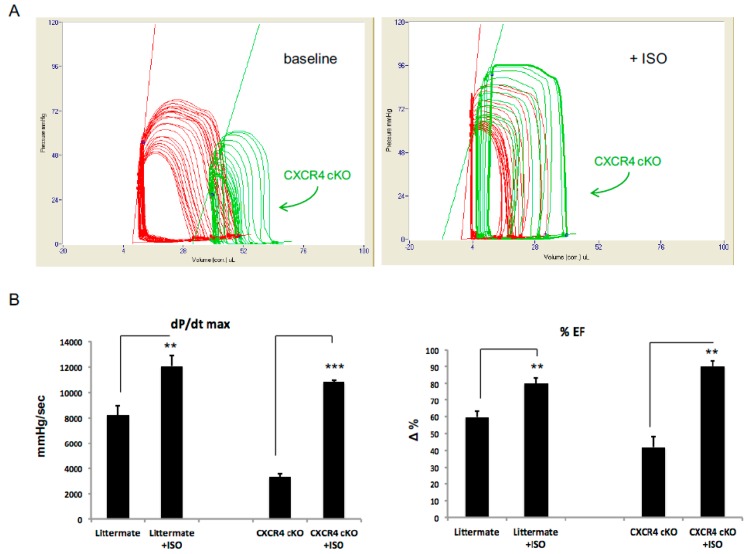
Effects of Isoproterenol in CXCR4 cardiac deficient mice on *in vivo* cardiac hemodynamics. (**A**) In vivo hemodynamic data that were acquired using a pressure-volume conductance catheter, via an open-chest approach, are shown at baseline and post ISO treatment (CXCR4 cKO-green and littermate WT-red). For this experiment we used mice at 4 months of age. We administered a 1 ng/g bolus of isoproterenol to the CXCR4 cKO, they responded in a very robust fashion overcoming their hemodynamic deficits and functioning similarly to WT controls, as shown by pressure-volume loop analysis. (**B**) Our data demonstrated that CXCR4 deficient mice had an augmented response to ISO with a greater increase in ejection fraction (EF) and dp/dt_max_. Importantly, since the CXCR4 cKO were operating at lower hemodynamic values, the exaggerated change in dP/dt_max_, and EF for experimental animals implies that CXCR4 deficiency confers additional sensitivity to isoproterenol (*n* = 5–7 mice/group) (** *p* < 0.01, *** *p* < 0.001).

Adrenergic receptors are involved in regulation of both physiologic and pathologic processes in the myocardium [24]. Our previous *in vitro* studies indicate that CXCR4 negatively modulates the ß-adrenergic response and the absence of CXCR4 may accentuate the ISO response [9,10]. Thus, we sought to investigate how cardiac-specific CXCR4 influenced the response to an acute challenge of ISO in vivo using a pressure-volume conductance catheter. For this experiment we used mice at 4-months of age, because this was the earliest time point that cardiac dysfunction was observed. We could not use mice at 12-months of age since they had severe clinical heart failure and were unable to complete the stress challenge secondary to high mortality. A PV catheter was inserted into the left ventricle via an open-chest, transapical stab and cardiac hemodynamic data were acquired. Once LV access was obtained, a 1 ng/g body weight bolus of ISO was administered via an external jugular venous cannula and hemodynamics were recorded at 1 min post-ISO infusion. Our data demonstrated that mice deficient in CXCR4 had an augmented response to ISO with a greater increase in ejection fraction (EF) and dp/dt_max_ (Figure 4B) (Table 2). This finding is a strong indicator how important the CXCL12/CXCR4 axis is in the modulation of contractility in response to adrenergic signals. Prior to isoproterenol infusion, there were significant baseline hemodynamic differences between the CXCR4 cKO in comparison to littermate controls (Figure 3B) (Table 2). However upon, ISO administration, the experimental animals demonstrated a robust response and overcame their baseline deficits to eventually reach WT values (Figure 3A,B) (Table 2). The exaggerated change in dP/dt_max_ for experimental animals implies that CXCR4 deficiency confers additional sensitivity to isoproterenol (Figure 4A,B). Table 2 delineates the left ventricular cardiac data recorded from WT and CXCR4 cKO mice (4-months). The effect of ISO; however, was only transient and the CXCR4 cKO mice cardiac function was back to the baseline within minutes. These in vivo hemodynamic findings, in conjunction with our MRI data, collectively reveal a significant decline in cardiac function in CXCR4 cKO mice, which worsens both: (1) As the mice age, and (2) under stress response conditions. Our findings further support the notion that CXCR4/CXCL12 plays an important, non-developmental role in the regulation of both baseline cardiac function as well as in response to β1-AR and β2-AR mediated stress pathways. 

## 3. Discussion

We present here a study characterizing the structural and functional implications of a cardio-selective CXCR4 knockout mouse line. The modulation of cardiomyocyte contractility is critical in preventing maladaptive remodeling and the development of heart failure. We have previously reported that overexpression of CXCR4 successfully prevented hypertrophy in trans-aortic constriction (TAC)-challenged mice [21] and that isoproterenol challenge in CXCR4 deficient mice resulted in cardiac hypertrophy [22]; these findings, in conjunction with the data presented in our current study suggest a powerful regulatory role for the CXCR4/CXCL12 axis. Deficient mice had progressed to clinical heart failure mediated by an increased susceptibility to isoproterenol-induced myocardial hypertrophy, increased hypertrophic marker expression, and increased interstitial fibrosis. This study advances the findings of our previous work by examining the effects of CXCR4 ablation on the normal murine life cycle in the absence of exogenous stress. CXCR4 expression was selectively abrogated in cardiomyocytes using a Cre-loxP-mediated gene deletion [22], and mice were allowed to live their normal life per our facility protocols. Non-invasive monitoring cardiac function was performed via gated cardiac MRI. During our observational period, mice typically met clinical endpoints by age 12-months and most died by 13-months. Given the invasive nature of hemodynamic measurement, mice were sacrificed following that procedure. Heart tissue samples were collected at various time points of the study for histological staining and biochemical analysis.

Based on our data, CXCR4 cKO mice showed mild cardiac dysfunction as early as 4-months of age, and by 12 months of age they had worsened fractional shortening and ejection fraction compared to wild-type mice, in addition to increased gross heart weight. By 6-months of age, CXCR4 cKO mice developed significant tissue fibrosis and our electron microscopy data revealed derangements in both the number and size of the mitochondria within CxCR4 cKO hearts. In addition to structural analysis, we completed functional analysis of CXCR4 cKO mice in both the presence and absence of exogenous isoproterenol. Prior to infusion of isoproterenol, we observed significant differences in baseline hemodynamics between CXCR4 cardiac deficient mice and wild-type controls; CXCR4 cKO exhibited lower systolic pressures, ejection fraction, and dp/dt_max_. Non-invasive MRI confirmed these results by demonstrating a decreased ejection fraction and larger end-diastolic and end-systolic volumes. These data suggest a mild cardiomyopathy in the CXCR4 cKO mice, even at early time points where there was minimal evidence of structural change. The mechanism by which CXCR4 knockout leads to decreased baseline performance is unknown and we continue to investigate the role of this axis in cardiac contractility. Interestingly, a 1 ng/g bolus of isoproterenol via transjugular catheter in the cKO group transiently corrected the baseline abnormality and the experimental animals overcame hemodynamic deficits and functioned similarly to WT controls, as shown by pressure-volume loop analysis. We postulate that the ISO challenge enhanced experimental animal ejection fraction by increasing contractility and thus improving cardiac output importantly, since the CXCR4 cKO were operating at lower hemodynamic values, the change in dp/dt_max_ and cardiac output were significantly greater than in the WT, which argues in favor of increased sensitivity to the isoproterenol. This enhanced response is maintained even when dp/dt was normalized against the LVPmax, which indicates an intrinsic enhancement of the CXCR4 cKO mice, by ISO.

Our group has previously reported that deletion of CXCR4 in cardiomyocytes exacerbates cardiac dysfunction following chronic isoproterenol exposure; *CXCR4 cKO* mice exposed to a 2-week infusion of exogenous catecholamine and isoproterenol demonstrated left ventricular wall thickening, chamber dilation, and reduced systolic function, as assessed by transthoracic echocardiography and invasive hemodynamic monitoring [22]. For that study, we chose knockout mice at 2 months of age who did not show histological abnormalities or structural deficits. However, in the catecholamine-induced stress state, these mice went on to cardiac dysfunction, exhibited lower systolic pressures, ejection fraction, and dp/dt_max_, as compared to controls, which was a strong indicator of how important the CXCL12/CXCR4 axis is in the modulation of contractility and heart pump function.

Altered α- and β-adrenergic receptor signaling is associated with cardiac hypertrophy and failure and β-blocker therapy is a mainstay of current clinical heart failure regimens. β-ARs play a fundamental role in heart failure transition and progression [25,26]. In the failure state, sympathetic overdrive and neural norepinehrine release in the myocardium maintains contractility and cardiac output due to reduced myocardial function [27]. Chronic β1-AR stimulation and Gs activity leads to receptor desensitization, internalization, and decreased inotropic reserve, along with activation of apoptotic pathways [28,29]. While this catecholamine response is important in maintaining systemic perfusion, the long-term implication is the progression of cardiac dysfunction and transition to heart failure hallmarked by a reduction in contractility and increased cell death. We have previously demonstrated the physical interaction and physiological association between β-adrenergic receptors and the chemokine receptor CXCR4 [9]. 

Others and we have shown that CXCR4, is known to regulate β-AR signaling in the cardiac myocyte by inhibiting L-type calcium channel activity [9,10]. An important transition in the development of CHF is the imbalance of calcium handling and defects in excitation-contraction coupling in the cardiac myocyte [30]. Thus, we postulated that the progression of heart failure in this model is associated with disturbances in the β-AR system and calcium cycling. The β-adrenergic signaling cascade serves as one of the most powerful regulatory pathways to enhance myocardial performance in response to systemic stress or increased demand for cardiac output. We developed this study to assess the functional and structural changes imparted by a loss of function in the CXCL12/CXCR4 signaling axis signaling in murine heart. We anticipated that, based on our present and previously published data, CXCR4 cKO animals would develop cardiac dysfunction and progress to clinical heart failure because of improper calcium handling secondary to inappropriate β-adrenergic activation. Catecholamine-induced cardiac hypertrophy and failure is associated with reduced contractile response to adrenergic agonists, an effect attributed to the down regulation of myocardial β-ARs, uncoupling of β-ARs and adenylate cyclase, as well as modifications of downstream cAMP-mediated signaling. 

We are aware that in our model of CXCR4 ablation, the CXCL12 alternative receptor, CXCR7, continues to function as an alternative receptor. We previously published that the ablation of CXCR4 has no effect on CXCR7 RNA or protein expression in this murine model [22]. Additionally, our group has shown that knockdown of both CXCR4 and CXCR7 in cardiomyocytes derived from human pluripotent stem cells differentially alters calcium transients and β-adrenergic responses, suggesting that these related receptors do have distinct functions [20]. Interestingly, the induced Pluripotent Stem Cells (iPSC) derived cardiomyocytes that contained knockdown of shCXCR4 showed a significant increase in chronotropy, as compared to scramble control, which further supports our observation here that the absence of CXCR4 causes dysfunction through adrenergic overactivation. This is in accordance with our previously published data indicating that CXCR4 is a negative modulator of β-ARs [9].

In parallel to catecholamine-induced injury in CXCR4 cKO animals, the mitochondrial derangements suggest that there are broad reaching effects from axis dysfunction. Mitochondria are the main intracellular location for fuel generation, and it is well described that mitochondrial dysfunction develops in the failing heart. We previously reported that CXCR4 ablation causes changes in mitochondrial function, oxidative stress, and biogenesis [22], which may correspond with the structural changes seen in our experimental animals. 

This study is the first to present a detailed characterization of the functional and structural changes seen in cardiac tissue derived from a murine CXCR4 cKO heart. We clearly demonstrate that there are derangements in the number and size of the mitochondrial within CXCR4 cKO hearts. Using a combination of MRI, hemodynamic, and histological analysis, we showed that CXCR4 cKO mice develop a progressive heart failure phenotype that leads to premature death. Our findings are a significant step forward in uncovering the post-developmental role of CXCL12/CXCR4; however, additional work is needed to better characterize the regulatory and signal modulating effects of cardiac CXCR4. These data suggest that the CXCR4 axis may serve as a future therapeutic target for patients whose contractile dysregulation will lead to a progressive cardiomyopathy and, ultimately, clinical heart failure.

## 4. Methods and Material

The investigation conforms to the Guide for the Care and Use of Laboratory Animals published by the US National Institutes of Health (NIH Publication No. 85-23, revised 1996).

### 4.1. Generation of CXCR4 Cardiac Specific Knockouts

Transgenic approaches are powerful tools to study gene function in development, physiology, and disease states. This is particularly important given that there are inadequate in vitro models to investigate the complex mechanisms of cardiac development and heart failure. However, the conventional use of tissue specific promoters, such as α-myosin heavy chain (αMHC) promoter [31], lacks temporal specificity in adult tissue and does not allow for the establishment of animal lines where transgene expression leads to a lethal phenotype [32]. We have developed a conditional knockout (cKO) cardiac myocyte-specific CXCR4-null mice (CXCR4-KO) that were obtained by cross-breeding CXCR4/loxP mice [33] (Jackson laboratory; Bar Harbor, Maine 04609) that contain loxP sites flanking CXCR4 exon 2 with mice carrying a transgene for Cre-recombinase under the control of the cardiac myocyte-specific α-myosin heavy chain promoter (αMHCre^+^) (courtesy of Dr. Michael Schneider, Baylor College of Medicine) [34]. Exon 2 of CXCR4 encodes 98% of the CXCR4 molecule. Cre recombinase-mediated deletion of exon 2 abolishes CXCR4 function. Littermates were tested by genotyping tail DNA [22]. 

### 4.2. Staining Procedures for Structural Abnormalities and Interstitial Fibrosis in Cxcr4 cKO Hearts

At the end of each time point, hearts were perfused with 30 mL of cold phosphate buffered saline with 0.1 mL of 1% heparin. LV was harvested, embedded in OCT (optimal cutting temperature), frozen, and stored at –80 °C. OCT heart samples were sectioned at 6–8 μm. Slides were then stained with hematoxylin and eosin (H&E) or Masson’s trichrome, as was previously described [22,35]. Briefly, Masson’s trichrome was conducted according to the guideline of the commercially available kit (Sigma, St, Louis, MO, USA). Frozen slides were fixed in Bouin’s solution for 15 min at 56 °C. Masson’s trichrome staining could be conveniently done on OCT samples with a 60 min incubation at room temperature followed by three washes. After Masson’s trichrome staining, distinct blue collagen fibers and red myocytes were observed in the tissues collected from hypertrophic hearts.

### 4.3. Real-Time Quantitative Reverse Transcription–PCR Assays

Whole ventricular tissues were minced and total RNA extracted using Trizol reagent (Gibco BRL, Carlsbad, CA, USA) according to manufacturer’s instructions. mRNA levels were determined by qRT–PCR using Clontech SYBR Advantage qPCR Premix (Clontech Inc., Mountain View, CA, USA). Primers were designed to generate short amplification products. The sequences of the specific primers were: ANF-Forward: 5’-CCTAAGCCCTTGTGGTGTGT-3’ and Reverse: 5’-CAGAGTGGGAGAGGCAAGAC-3’; 28SrRNA-Forward: 5’-CTCGCTGGCCCTTGAAAATCC-3’; and Reverse: 5’-CCCAGCCCTTAGAGCCAATCCTTA-3’. Reverse transcription was performed using a kit according to manufacturer’s instructions (Applied Biosystems, Foster City, CA, USA). Real-time PCR was performed in 10 μL reaction volumes using 10 pmol of primers. The relative levels of gene expression were calculated according to the manufacturer’s recommendations. Ribosomal 28S RNA was used as an internal control to calculate the relative abundance of mRNAs. 

### 4.4. Transmission Electron Microscopy

Cardiac tissue was incubated overnight in Karnovsky’s fixative and this was then replaced with Karnovsky’s storage buffer. Several 1 mm^3^ pieces of heart wall were transferred to 70% ethanol and embedded in plastic resin. One-micrometer-thick sections were prepared on an ultramicrotome glass and stained with toluidine blue. The thin sections were stained with uranyl acetate and then with lead citrate and examined with a Zeiss 910 electron microscope (Seattle, WA, USA). Random pictures of each section were taken at 3000–5000× magnification for the mitochondrial count and at 1000× magnification for the dark and light fiber count. All preparation and imaging of the samples were performed at the Electron Microscopy Core Facility at Icahn School of Medicine at Mount Sinai under supervision of Dr. Ron Gordon.

### 4.5. Non-Invasive Cardiac Imaging: Gated Cardiac Magnetic Resonance Imaging (MRI)

MRI experiments were performed using a General Electric Omega 9.4T vertical bore MR system equipped with a micro-imaging accessory and custom-built coils designed specifically for mice, as described previously [36]. Just prior to each image acquisition, the heart rate was determined from the electrocardiogram, and the spectrometer gating delay was set to acquire data in diastole and systole. Multi-slice spin-echo imaging with an echo time of 18 ms and a repetition time of ∼100–200 ms was performed. A 35 mm field of view (with a 256 × 256 pixel image matrix) was used. Short and long axis images of the heart were acquired, and MRI data were processed off-line with MATLAB-based custom-designed software. 

### 4.6. In Vivo Hemodynamic

In vivo hemodynamics were performed using a 1.2 Fr pressure-volume (PV) conductance catheter (Scisense, Ontario, Canada). Mice were anesthetized with an intraperitoneal injection of urethane (1 mg/g), etomidate (10 μg/g), and morphine (1 μg/g) and intubated via a tracheotomy and mechanically ventilated at 7 μL/g tidal volume and 125 respirations/minute. The PV catheter was placed in the left-ventricle via an apical stab approach as previously described [21,22]. Hemodynamic data were obtained at pre- and post-isoproterenol administration. 1 ng/g bolus of ISO was administered via an external jugular venous cannula and hemodynamics were recorded at 1 min post-ISO infusion. Pressure-volume data were analyzed using IOX2 software (EMKA technologies).

### 4.7. Statistical Analyses

Numeric data are presented as mean ± S.E.M. Student’s *t*-test and one-way analysis of variance (ANOVA) with Tukey’s post hoc test was performed between all groups at each time point. Student’s *t*-test were also utilized where it was applicable with *p*-values <0.05 considered statistically significant (* *p* < 0.05, ** *p* < 0.01, ^#^ *p* < 0.001).

## Figures and Tables

**Table 1:** MRI baseline analysis at 4 months of age. MRI experiments were performed using a General Electric Omega 9.4T vertical bore MRI system equipped, with a micro-imaging accessory and custom-built coils designed specifically for mice. MRI was performed at 4 months of age to analyze the baseline cardiac function. At 4 months of age, the CXCR4 cKO group demonstrated a significantly lower ejection fraction, as well as an increase in end-diastolic and end-systolic volumes suggesting mild functional defects. These data seem to suggest a mild cardiomyopathy present in the CXCR4 cKO. Abbreviations: EF, ejection fraction; EDV, end-diastolic volume; ESV, end-systolic volume; HR, heart rate.

Item	Litermate WT	stdev	CXCR4 cKO	stdev
Ejection Fraction (%) *	79.41875	9.492518012	68.04285714	5.701349882
ED volume (µL) *	22.6825	6.270105376	28.19714286	4.227228068
ES Volume (µL) *	4.8	3.195000559	9.088571429	2.392247399
ED Volume Index (µL/g)	0.9225	0.273663419	1.127142857	0.168593622
ES Volume Index (µL/g)	0.2525	0.155857841	0.364285714	0.096411815
Stroke Volume (µL)	17.88	4.868516935	19.10714286	2.815402365
Stroke Volume Index (µL/g)	0.675	0.155026879	0.764285714	0.112673147
Cardiac Output (ml/min)	6.2225	1.05787129	6.835714286	2.328482032
ED Myocard Mass (mg)	46.14625	10.08292607	44.24142857	5.795801436
ES Myocard Mass (mg)	48.625	6.825544458	50.58	6.131728957
Heart Rate (bpm)	375	50	360	111.9523708

Values are means ± SE; Intragroup; * *p* < 0.05.

**Table 2:** In vivo hemodynamics: Pressure-volume data were analyzed using IOX2 software. The PV catheter was inserted into the left ventricle via an open-chest, transapical stab and cardiac hemodynamic data was acquired. For this experiment we used mice at 4 months of age. Prior to infusion of isoproterenol, we observed significant differences in baseline hemodynamics between CXCR4 cardiac deficient mice and wild-type controls. The CXCR4 cKO exhibited lower systolic pressures, ejection fraction, and dp/dt_max_ as compared to controls. Our data demonstrated that CXCR4 deficient mice had an augmented response to ISO with a greater increase in ejection fraction (EF) and dp/dt_max_. Abbreviations: LVP, left ventricular pressure; CO, cardiac output; SV, stroke volume; EF, ejection fraction.

Item	Littermate WT (4-Month)		CXCR4 cKO (4-Month)	
	Baseline	ISO, 1 min	Baseline	ISO, 1 min
Heart Rate, bpm	548 ± 17	618 ± 15 *	510 ± 8.5	604.3 ± 22.4 *
LVP_max_, mmHg	97.5 ± 3.3	95.3 ± 5.1	64.3 ± 4.6	95.3 ± 9.3 *
Pes, mmHg	88.8 ± 3.8	87.1 ± 2.3	55.7 ± 5.5	72.0 ± 13.6
Ped, mmHg	3.5 ± 0.5	5.0 ± 0.5	2.6 ± 0.3	1.2 ± 0.2
dP/dT_max_, mmHg/sec	8192 ± 701	12060 ± 868 **	3322 ± 253.2	10780 ± 121.9 ***
dP/dT_max_/LVP_max_, s^−1^	90.1 ± 5.8	127.1 ± 5.3 **	51.7 ± 2.6	115.3 ± 11.2 **
CO, uL/min	16000 ± 1290	22630 ± 1982 *	12270 ± 1840	20850 ± 1539 *
SV, uL	29 ± 1.8	38.2 ± 2.7 *	24.0 ± 3.5	34.7 ± 3.3
EF, %	59.8 ± 3.5	80.2 ± 3.2 **	42.0 ± 6.1	90.3 ± 2.9 **
Ved, uL	47.3 ± 2.1	44.2 ± 2.7	55.7 ± 0.3	38.3 ± 2.9 **
Age, weeks	11.5		12.1	
Weight, g	26.2		26.1	

Values are means ± SE; Intragroup, * *p* < 0.05, ** *p* < 0.01, *** *p* < 0.001.

## Supplementary Materials

Supplementary materials can be found at https://www.mdpi.com/1422-0067/20/9/2267/s1.
