# Supplementary material for: CXCR4 Cardiac Specific Knockout Mice Develop a Progressive Cardiomyopathy
Source: Int J Mol Sci. 2019 May 8;20(9):2267. doi: 10.3390/ijms20092267 (PMC6539363; doi:10.3390/ijms20092267)
Supplement: Supplementary file 1 [file ijms-20-02267-s001.pdf]

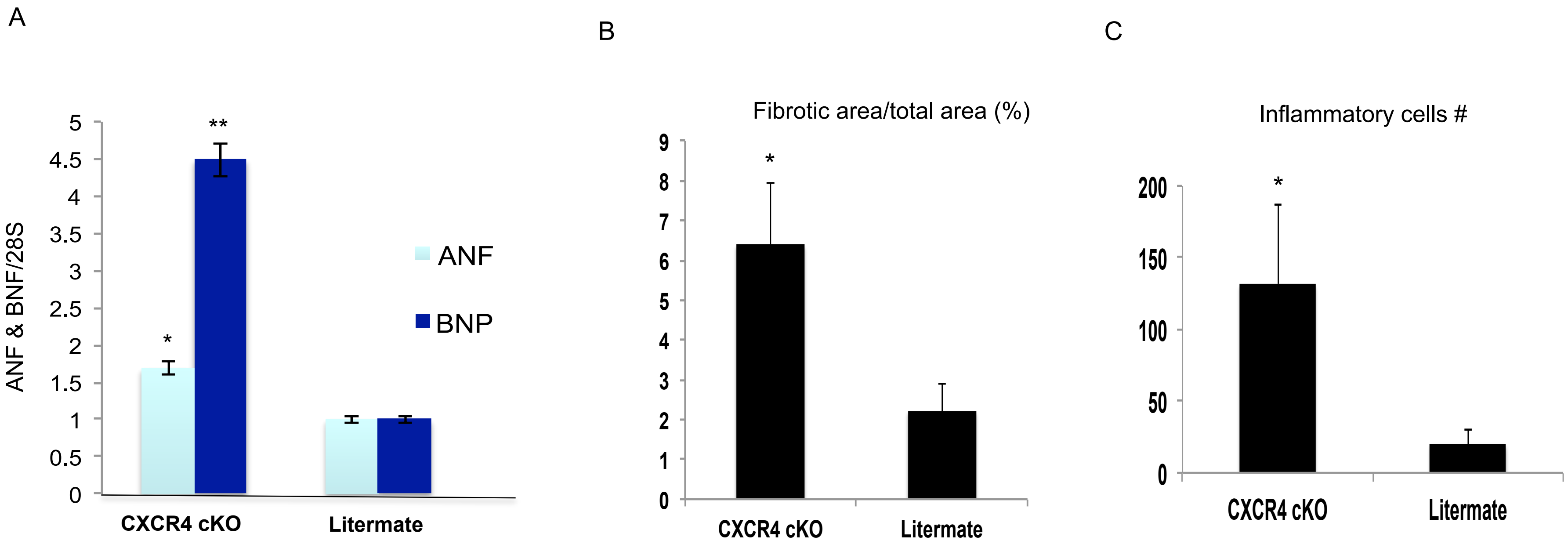

Figure S1: Histological analysis performed on hearts obtained from CXCR4 cKO and littermate control at 6 months of age. A) RNA was isolated from whole ventricular myocardium and the expression of hypertrophy associated genes e.g. ANP and BNP were assessed. Specific mRNA levels were quantified via qRT-PCR performed in triplicate. The significance of each group is shown as CXCR4 cKO was compared to littermate control group (n=3 mice/group *=P<0.05). B) The number of inflammatory cells were quantified at 40X and average cell count was graphed (n=3 mice/group *=P<0.05). C) Quantification of fibrosis was calculated over the entire section on the images taken at 40X magnification. The significance of each group is shown as CXCR4 cKO was compared to littermate control group (n=3 mice/group *=P<0.05).
